# Evolving Bacterial Envelopes and Plasticity of TLR2-Dependent Responses: Basic Research and Translational Opportunities

**DOI:** 10.3389/fimmu.2013.00347

**Published:** 2013-10-28

**Authors:** Junbin Li, Dennis Sang Won Lee, Joaquín Madrenas

**Affiliations:** ^1^Microbiome and Disease Tolerance Centre, Department of Microbiology and Immunology, McGill University, Montreal, QC, Canada

**Keywords:** toll-like receptors, gram-positive bacteria, commensal bacteria, microbiota, immune response

## Abstract

Innate immune mechanisms that follow early recognition of microbes influence the nature and magnitude of subsequent adaptive immune responses. Early detection of microbes depends on pattern recognition receptors that sense pathogen-associated molecular patterns or microbial-associated molecular patterns (PAMPS or MAMPs, respectively). The bacterial envelope contains MAMPs that include membrane proteins, lipopeptides, glycopolymers, and other pro-inflammatory molecules. Bacteria are selected by environmental pressures resulting in quantitative or qualitative changes in their envelope structures that often promote evasion of host immune responses and therefore, infection. However, recent studies have shown that slight, adaptive changes in MAMPs on the bacterial cell wall may result in their ability to induce the secretion not only of pro-inflammatory cytokines but also of anti-inflammatory cytokines. This effect can fine-tune the subsequent response to microbes expressing these MAMPs and lead to the establishment of a commensal state within the host rather than infectious disease. In this review, we will examine the plasticity of Toll-like receptor (TLR) 2 signaling as evidence of evolving MAMPs, using the better-characterized TLR4 as a template. We will review the role of differential dimerization of TLR2 and the arrangement of signaling complexes and co-receptors in determining the capacity of the host to recognize an array of TLR2 ligands and generate different immune responses to these ligands. Last, we will assess briefly how this plasticity may expand the array of interactions between microbes and immune systems beyond the traditional disease-causing paradigm.

## Introduction

Innate immunity is characterized by an immediate response against pathogens and is paramount in the initial control of infection. Toll-like receptors (TLRs) are thought to be among the most ancient pathogen recognition systems (1). Such systems recognize pathogen- or microbial-associated molecular patterns (PAMPs or MAMPs) that are relatively conserved among microorganisms but are absent in hosts. This recognition involves pattern recognition receptors (PRRs) that are broadly expressed by a variety of immune cells such as monocytes, macrophages, dendritic cells (DCs), and some epithelial cells. PRR-dependent responses are critical for promoting inflammatory processes leading to microbial clearance.

The genes encoding receptors of the innate immune system are inherited and not subject to gene rearrangement like those coding for adaptive immune receptors. As a result, the innate response has been traditionally considered fairly static in its recognition of microbial patterns, and to be largely indiscriminate in promoting inflammation and priming adaptive immunity. However, emerging evidence indicates that PRR signaling can also promote anti-inflammatory responses under certain conditions (2–5). The precise role of PRR-induced anti-inflammatory responses in the context of innate immunity remains uncertain. Recent evidence suggests that these anti-inflammatory responses may play an important role in the regulation of the homeostatic tolerance to commensal organisms or uncontrolled microbial proliferation (6).

Two views of the biology of PRR-induced responses may be considered. One is that host responses to MAMPs vary depending on the context of exposure (e.g., site, responding cell type, expression of co-receptors by responding cell, etc.), which ultimately determines the type of response (pro- vs. anti-inflammatory responses). Alternatively, we should not forget that MAMPs are subject to evolutionary modification as a result of selective pressures by host defense mechanisms, and that this evolution will ultimately expand the array of ligands to a given PRR and the signals that it can deliver. However, co-evolution of both MAMPs as well as host responses can explain the two opposite outcomes of PRR signaling. In this review, we will focus only on TLRs, and specifically on TLR2 and TLR4, to review the plasticity of signaling by these receptors and its biological implications.

## Overview of Toll-Like Receptors

Toll-like receptors were the first class of PRRs identified. In 1989, Charles Janeway hypothesized that the innate immune system senses the presence of microbes by recognizing commonly associated molecular signatures (7). In 1996, his hypothesis was vindicated with the discovery of the Toll pathway in *Drosophila* and its role in controlling fungal infections (8) and subsequently with the characterization of associated PRRs Gram-negative bacteria binding protein 3 (GNBP3) and peptidoglycan (PGN) recognition protein SA (PGRP-SA), which recognize bacterial and fungal MAMPs (9, 10). Soon after, Medzhitov et al. (11) discovered a human homolog for Toll, at the time termed hToll, which was found to activate the transcription factor NF-κB. Mice deficient in this receptor were unable to induce pro-inflammatory cytokines to lipopolysaccharide (LPS) (12). This receptor, later called TLR4, was the first member of the TLR family of receptors to be characterized, a family that now includes 13 members, 10 of which are expressed in humans (13).

Structural studies have characterized TLRs as type I transmembrane proteins with leucine-rich repeats (LRRs) on the extracellular N-terminal domain (13). This LRR domain contains an α-helix and a β-strand linked by loops, leading to the prediction that the ectodomain of TLRs assume a horseshoe conformation (14). The intracellular C-terminal portion of TLRs contains a Toll/interleukin-1 receptor (TIR) domain, which is common to all members of the TLR family (15).

Toll-like receptors are constitutively expressed on monocytes, macrophages, and DCs, while certain TLRs are also expressed on other cell types such as neutrophils, mast cells, epithelial cells, and B cells (13). TLR1, 2, 4, 5, 6, and 10 are expressed on the cell surface, while TLR 3, 7, 8, and 9 are expressed in intracellular compartments such as the endosome, lysosome, and the endoplasmic reticulum (1). TLRs 1–9 are conserved between humans and mice. TLR10 is a non-functional pseudogene in mice due to a retroviral insertion, but is nonetheless a functional receptor in humans (16). TLRs 11, 12, and 13 are poorly characterized and are absent from the human genome (17).

Through TLRs, the host can recognize a wide variety of microbial ligands including nucleic acids, lipids, lipoproteins, and polysaccharides. TLRs can be grouped according to their recognition of similar MAMPs. For example, TLRs 3, 7, 8, 9, and 13 all recognize nucleic acids. However, TLRs can also recognize a repertoire of structurally unrelated ligands. TLR4 has long been recognized as the receptor for LPS, but also recognizes heat-shock proteins (18), glycoproteins such as fibronectin (19), the fusion protein of respiratory syncytial virus (RSV) (20), and the plant diterpene and chemotherapy drug paclitaxel (21, 22). Similarly, TLR2 can recognize lipoteichoic acid (LTA), PGN, lipopeptides, and zymosan (reviewed in Ref. (13) and see below). Unlike TLR4 which signals as a homodimer, TLR2 forms heterodimers with TLR1, TLR6, and perhaps TLR10, hinting at a potential mechanism to discriminate microbial ligands and elicit varied downstream responses.

Toll-like receptors function as important immune receptors that, in coordination with other PRRs, turn on innate mechanisms of immunity, including inflammation. It is thus expected that changes in TLR ligand binding and signaling capacity will translate in changes in innate immunity. This is well illustrated by observations that certain polymorphisms in TLRs have been associated with increased susceptibility to a myriad of infectious diseases (23, 24) and to some non-infectious diseases such as cancer (25–27).

## Molecular Basis of TLR2 and TLR4 Signaling

Since the discovery of TLRs, signaling from these receptors has been recognized as potently eliciting pro-inflammatory responses in the host (15). The bacterial endotoxin LPS found in the Gram-negative bacterial cell wall, and lipopeptides and glycopolymers in the PGN layer of Gram-positive bacterial cell wall are potent stimulants of inflammation, following TLR4 and TLR2 recognition respectively. PGN can stimulate inflammation via other PRRs such as nucleotide-binding oligomerization domain-containing protein (NOD1) and NOD2. Inflammation is characterized by the production of a vast array of cytokines including IL-1β, IL-6, and TNFα, as well as chemokines such as IL-8 and MCP-1/CCL-2, and type I interferons (IFN) through the NF-κB and IRF3 transcription factors. The observation that down-regulation of TLR2-mediated signaling with transmembrane domain-derived peptides reduces lethality in mice following intraperitoneal challenge with *Staphylococcus aureus* (28) illustrates that these pro-inflammatory mediators may be deleterious to the host when left unchecked.

Signaling from TLR2 and TLR4 is initiated by their ligand-induced dimerization. This step brings the TIR domains in their cytoplasmic tails into close proximity, forming a platform for signaling through TIR domain-containing adaptor molecules. TLR2 and TLR4 are phosphorylated on tyrosine residues, and deficiencies in their phosphorylation are associated with defective dimerization and impaired recruitment of TIR domain-containing adaptors (29, 30). Five TIR domain-containing adaptors have been identified: MyD88, MAL/TIRAP, TRIF, TRAM, and SARM [reviewed in Ref. (31)]. MyD88 was the first such adaptor discovered, and is involved in the signaling of all the TLRs except TLR3. TLR4 is known to utilize two distinct signaling pathways (MyD88-dependent and MyD88-independent pathways), mediated by different TIR domain-containing adaptor molecules, and leading to the induction of pro-inflammatory cytokines through mitogen-activated protein (MAP) kinase and NF-κB activation. However, MyD88-deficient mice challenged with LPS were still able to induce the activation of NF-κB and JNK (a MAP kinase) with a delayed response (32). This MyD88-independent, TRIF-dependent pathway was later shown to induce pro-inflammatory cytokines as well as type I IFN through IRF3 (33). TRIF was found to interact with both TLR3 and TLR4, but TRIF-deficient mice did not produce type I IFN in response to TLR3 or TLR4 ligands (34, 35). In contrast to TLR4, TLR2 mostly depends on the adaptors MyD88 and TIRAP/MAL for signaling (36, 37). Mice deficient in MAL displayed a similar phenotype to MyD88 knockout mice when challenged with LPS, exhibiting delayed activation of NF-κB and MAPKs (37). MAL is thought to be particularly important for TLR2 signaling, as activation of NF-κB and MAPKs is impaired to a greater extent in MAL-deficient cells following PGN stimulation compared to LPS stimulation (36). Additional roles for cell-type-specific TLR2 adaptors have been reported. For example, peritoneal but not bone-marrow-derived macrophages can produce IFN-β through IRF3 and IRF7 in manner independent of MAL but dependent on TLR2 and TRIF (38).

## Differential Signaling by Gram-Negative Bacteria: An Indication for Evolving Innate Immune Recognition

Lipopolysaccharide is the major component of the outer leaflet of the Gram-negative cell envelope (12, 39, 40) and as such is the most abundant Gram-negative MAMP capable of inducing an immune response. LPS is composed of an O-antigen polysaccharide chain, an oligosaccharide core, and a lipid A anchor (41). The O-antigen polysaccharide projects away from the bacterial cell and its structure is highly variable from strain to strain. The oligosaccharide core component serves as a linker between the O-antigen and the lipid A anchor, which is the primary mediator of LPS toxicity through TLR4-mediated recognition and signaling, causing fever, diarrhea, and septic shock (42).

Lipid A, however, is not absolutely conserved across species. Hexa-acylated forms of lipid A found in *Escherichia coli* and *Salmonella enterica* serovar Typhimurium are potent activators of TLR4 (43, 44). This pro-inflammatory response is demonstrated by the production of IL-1β, IL-6, IL-12p40, and TNFα by monocytes and DCs leading to the organization of an inflammatory response at the site of infection (42). Conversely, tetra-acylated forms found in *Helicobacter pylori* and some forms of *Pseudomonas aeruginosa* do not activate TLR4 to a similar extent (45–47). Additionally, LPS species from *Porphyromonas gingivalis* induce TNFα and IL-1 but not IL-12 and IFN-γ via a TLR2-mediated mechanism, and LPS from *Rhodobacter sphaeroides* does not induce pro-inflammatory cytokine production (48). These species- and/or strain-specific differences point to diversity in TLR4 ligands as well as the versatility of innate immune receptors in cross-recognition of diverse MAMPs. Such diversity and versatility is consistent with the evolving nature of the bacterial cell wall.

In addition to differences in stimulatory capacity of LPS between bacterial strains, some microbes alter their lipid A moieties in response to environmental cues, a change that also translates into diversity in host recognition and response (49). This phenomenon is well documented for the PhoP/PhoQ two-component system in *Salmonella* spp. (50). PhoQ, located on the inner membrane, is activated by anti-microbial peptides, low magnesium concentrations, and/or acidic pH (51). Following activation, PhoQ phosphorylates and activates the transcriptional regulator PhoP. One of PhoP’s effector functions results in the modification of lipid A by the 3-*O*-deacylase, PagL, and a palmitoyl transferase, PagP. PagL modifies lipid A by removing one fatty acid chain and creating a penta-acylated lipid A (52, 53). In contrast, PagP modifies lipid A by adding an additional fatty acid chain, creating a hepta-acylated lipid A. When both enzymes modify a lipid A moiety, the end result is a hexa-acylated lipid A with one less acyl chain linked to the core region and an additional acyl chain linked via another fatty acid. Kawasaki et al. (53) identified that the lipid A products of PagL and/or PagP led to a decrease in TLR4-mediated NF-κB activation. This PhoP/PhoQ two-component system identifies just one example of a modification in *S. enterica* lipid A that modulates the innate immune response. This system is thus a good example to illustrate the evolving nature of the bacterial cell wall.

Lipopolysaccharide has also been reported to induce the production of the anti-inflammatory cytokine IL-10 in co-cultures of T cells with stimulated DCs as well as in primed DCs (54). For example, LPS from *Pseudoalteromonas* strains was able to modulate the pro-inflammatory response to *E. coli* LPS owing to a combination of low immunostimulatory activity as well as competition for TLR4 (55). Co-stimulation with the two types of LPS resulted in lower pro-inflammatory cytokine production and higher IL-10. This indicated an additional potential role for LPS from commensal bacteria in maintaining immune homeostasis and preventing inflammatory diseases.

The evidence discussed above suggests that a single MAMP from different species can take on various forms and possess different immunostimulatory capacity via the same PRR based on modifications induced by environmental stimuli. Furthermore, a potential regulatory role may be played by LPS with regards to competition for TLR4 and induction of IL-10 in a mechanism that has yet to be elucidated. Experimental models of inflammatory bowel disease (IBD) have helped to identify genetic factors that reveal host susceptibilities to impairment of gut immune homeostasis (38). However, the products and components of the microbiota, such as LPS, may play a larger role in setting the balance of organisms in a given microbiota and the maintenance of the local immune environment (6, 56).

## Anti-Inflammatory TLR4 Signaling

**Figure 1:**
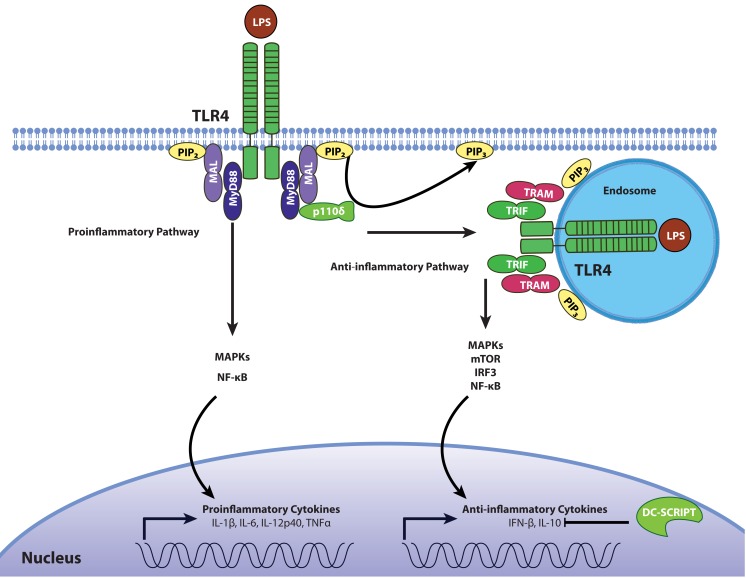
**Regulation of pro- and anti-inflammatory TLR4 signaling**. In the resting state, phosphatidylinositol on the cell surface exists mostly as PIP_2_, allowing the recruitment of MAL/TIRAP which contains a PIP_2_ binding domain. Following stimulation with LPS, TLR4 activation leads to the recruitment of adaptors TIRAP/MAL and MyD88 during early stages of MAMP recognition to promote the production of inflammatory cytokines through the MAP kinases JNK and p38 as well as the NF-κB transcription factor. The phosphorylation of PIP_2_ into PIP_3_ by the p110δ isoform of PI3K causes the translocation of the receptor-ligand complex into endosomal compartments, where TLR4 signals through the adaptors TRIF and TRAM to induce the secretion of IL-10 and type I IFNs through MAP kinases, NF-κB, and IRF3. This mechanistic framework is likely operational in macrophages but not in DCs depending on expression of DC-SCRIPT, a transcription factor which suppresses IL-10 expression.

Although the pro-inflammatory effects of TLR4 signaling have been described in detail, the anti-inflammatory responses induced by TLR4 activation are much less characterized. The cell compartment where TLR4 signaling occurs seems to be an important factor when it comes to differentiating between pro- and anti-inflammatory signaling. Contrary to TLR4 pro-inflammatory signaling at the cell surface, TLR4 signaling from endosomal compartments induces the secretion of the anti-inflammatory cytokine IL-10 (2). This compartment-dependent effect is due to a shift from MyD88/MAL-dependent pro-inflammatory signaling at the cell surface to TRIF/TRAM-mediated anti-inflammatory signaling in the endosome, and is mediated by the p110δ isoform of PI3-kinase (PI3K). This PI3K isoform is involved in the TLR4 internalization, dissociation of MAL from the cell membrane, and subsequent degradation of MAL by calpain (2). Therefore, a shift in adaptor molecule use is associated with a shift in cytokine expression (Figure 1): the MyD88-dependent pathway leads to the production of inflammatory cytokines such as IL-1β, IL-6, and TNFα, whereas the TRIF-dependent pathway induces IFN-β and IL-10. This model is corroborated by the observation that inactivation of p110δ results in higher levels of the pro-inflammatory cytokines and lower levels of IFN-β and IL-10.

Other signaling events downstream of TLR4 activation leading to this anti-inflammatory response are unclear. The MAP kinases p38 and JNK have been shown to be necessary for IL-10 secretion by bladder epithelial cells (BECs) (57). Inhibition of p38 and JNK in LPS-activated BECs reduces STAT3 expression, and this enhances IL-10 secretion (58). Therefore, the balance between pro- and anti-inflammatory TLR4 signaling may depend not only on the use of either the MyD88/MAL or TRIF/TRAM adaptor proteins respectively, but also on additional factors. TRIF also plays a role in pro-inflammatory signaling, as evidenced by the complete abrogation of LPS-stimulated NF-κB and JNK activation in MyD88 and TRIF double knockout mice (35) but not in mice deficient for MyD88 alone, although the latter have delayed kinetics compared to wild-type control mice (32). Furthermore, quantitative proteomic analysis of the secretome of LPS-activated murine bone-marrow-derived macrophages suggests that both MyD88 and TRIF are required for full IL-10 expression, since IL-10 secretion was at least 100-fold greater when both adaptors were present compared to conditions in which only one was present (59).

## An Expanding Array of TLR2 Ligands

TLR2, in conjunction with other TLRs (see below), has been identified as a receptor for an array of ligands including PGN-embedded LTA, di- and tri-acylated lipopeptides, lipoproteins, and others including LPS variants (60, 61), zymosan from fungi, lipoarabinomannan from mycobacteria, glycosylphosphatidyl inositol mucin from *Trypanosoma cruzi*, hemagglutinin protein from measles virus, and phospholipomannan from *Candida albicans* [reviewed in Ref. (13)]. The envelope of Gram-positive bacteria contains a multitude of molecules that can act as MAMPs. These include the glycopolymers wall teichoic acid (WTA) and LTA, PGN fragments, lipopeptides and lipoproteins, and other proteins (40).

For years, LTA has been touted as an LPS equivalent for Gram-positive bacteria by acting through a TLR2-mediated mechanism. LTA is a structural glycopolymer within the cell wall that is anchored to the cell membrane but protrudes outward through the cell wall (40). It is constituted of a polymer of repetitive 1,3-phosphodiester-linked glycerol-1-phosphate units with a glycolipid anchor. Some glycerol phosphate subunits are substituted with d-alanine residues, and are responsible for the stimulating properties of this molecule when tested with synthetic LTA (62). This substitution is also partially responsible for the resistance of some Gram-positive species to anti-microbial peptides (63). The glycolipid anchor often contains two acyl chains that may be responsible for binding to TLR2 (62). The precise contribution of LTA to the overall TLR2-triggered response is uncertain (64). It has been reported that only lipoproteins and lipopeptides can induce TLR2 signaling under physiological concentrations and that other cell wall fractions (such as the LTA fraction) are contaminated by these molecules (64, 65).

Although both lipoproteins and PGN are found in both Gram-negative and Gram-positive species, the magnitude of LPS-TLR4 interaction often overshadows the immunostimulatory capacity of lipoproteins in Gram-negative bacteria. Although lipoproteins are likely a primary bacterial ligand for TLR2 (64), most experimental data on TLR2 function has been generated using synthetic lipopeptides. Structural studies using the synthetic lipopeptides Pam2CSK4 and Pam3CSK4 show that TLR2 dimerized with TLR1 recognizes triacylated lipoproteins while TLR2–TLR6 dimers recognize diacylated lipoproteins (66, 67). This functional distinction aligns with the structural conformation observed from the crystal structures of these heterodimers (66). Intriguingly, the acyl chains of the lipoproteins, which are hypothesized to bind to TLRs, are embedded into the bacterial membrane, making it uncertain whether they are accessible to immune receptors. In Gram-positive bacteria, the lipoproteins are found below the PGN wall but on the outer leaflet of the cytoplasmic membrane (40). In Gram-negative bacteria, the lipoproteins are found on the outer leaflets of the inner and outer membranes. However, Boneca et al. (68) have shown that phagocytosis and proteolytic destruction of *S. aureus* by macrophages may be necessary for TLR-mediated responses and in this way contribute to the generation of ligands for TLR2 or NOD receptors that amplify the innate response to these bacteria. Furthermore, some triacylated lipoproteins have been found to induce an immune response dependent on TLR2 but independent of TLR1 or TLR6 (69). Of interest, environmental conditions may impact the balance between di- vs. tri-acylated lipopeptide expression by bacteria and, thus influence the type of ensuing responses (70). Gram-positive bacteria can also modify their MAMPs in response to selective pressures applied by the host immune system. *Listeria monocytogenes* peptidoglycan *N*-deacetylase (PgdA) decreases its recognition by innate immune receptors, playing a role in immune evasion. Strains with this gene knocked out displayed increased sensitivity to lysozyme, impaired survival in macrophages, and impaired virulence *in vivo* in BALB/c and C57/BL6J mice (68). Based on the heterogeneity of TLR2 ligands, it is plausible to suggest that there may be an array of ligands for TLR2 that results from environmental selection upon microbial-host interactions. If this is the case, then fine characterization of mechanisms involved in TLR2-mediated recognition and signaling may turn out to be a much more laborious exercise than initially thought.

## Plasticity in TLR2 Signaling

As mentioned above, the MAMPs that can act as TLR2 ligands (in association with TLR1, TLR6, and maybe TLR10) are many and structurally diverse, albeit sharing lipid moieties (13). The variation in structure and biological origins suggest that there is a considerable plasticity in TLR2-dependent recognition and signaling. Indeed, like TLR4, TLR2 has recently been recognized as capable of eliciting anti-inflammatory cytokine responses (3–5). For example, upon stimulation with staphylococcal PGN preparations, peripheral blood mononuclear cells (PBMCs) produced IL-10 through a TLR2-dependent mechanism, down-regulating the T cell response to staphylococcal superantigens (69). Further studies indicated that the PI3K-Akt pathway was indispensible for IL-10 production, as the IL-10 response was associated with Akt phosphorylation and was inhibited by the PI3K inhibitor wortmannin (71).

Unlike TLR4 which signals as a homodimer, TLR2-dependent MAMP recognition and signaling requires, under most circumstances, formation of TLR2 heterodimers. TLR2 is thought to exist in pre-formed low affinity complexes associated with TLR1 and TLR6 under basal conditions and dimerizes upon ligand binding (66), heterodimerizing with TLR1 or TLR6 upon recognition of triacylated and diacylated lipopeptides respectively (72, 73). Such a heterodimerization of TLR2 has been considered a factor potentially determining the ensuing pro- vs. anti-inflammatory responses. In general, TLR2/1 complexes have been more often linked with pro-inflammatory responses than TLR2/6 complexes, which have been linked with anti-inflammatory responses (4, 74). The structural basis for this difference is unknown at the moment. In addition, it has been claimed that TLR2 homodimers may down-regulate TLR2-dependent responses. However, the evidence for this claim is solely based on *in vitro* recombinant systems, using chimeric proteins of the extracellular domain of CD4 fused with the transmembrane and intracellular domains of TLR2, and may not take into account other regulatory factors associated with natural ligand-induced TLR2 dimerization (72).

Other cell surface PRRs that could associate with TLR2 can also contribute to the different functional outcomes of TLR2 engagement. SitC, a triacylated lipoprotein found in *S. aureus*, was recently shown to induce IL-6 and TNFα through TLR2 and MyD88 in peritoneal macrophages (75). This was observed even in mice deficient in TLR1 and TLR6, suggesting that TLR2 can signal through dimers other than TLR2/1 and TLR2/6, either TLR2 homodimers or, alternatively, TLR2 dimerizing with other PRRs. In human cells, one such candidate could be TLR10. Although not expressed in mouse cells, TLR10 is a functional MyD88-dependent receptor in humans, being expressed on B cells and certain DC subsets (16). In 2001, TLR10 was identified as a receptor with the characteristic LRRs and Toll/IL-1 receptor TIR domains shared by all TLRs (76). Importantly, this study found that TLR10 was highly homologous to TLR1 and TLR6 with an overall amino acid identity of 50 and 49% respectively, whereas it was only 30% identical to TLR2 and no more than 25% for the other TLRs. Phylogenetic studies on TLR evolution indicate that TLR10 predates TLR1 and TLR6, suggesting that TLR1 and TLR6 arose from gene duplication, consistent with the observation that TLR10, TLR1, and TLR6 lie in tandem on human chromosome 4 (77). It has been suggested that TLR10 shares ligand recognition with TLR1 but signals differently (78). Given that TLR10 polymorphisms have been associated with various human pathologies (79–81), TLR10 represents a promising direction in which to increase our understanding of the plasticity of TLR2 responses.

Another factor to consider in the signaling properties of TLR2 is the availability of co-receptor molecules. Among these, CD14 (82) and CD36 (83) are accessory molecules known to contribute to certain responses to TLR2 signaling [reviewed in Ref. (84)]. Availability of these molecules is important for pro-inflammatory responses to TLR2 ligation but not for IL-10 production (71). These accessory proteins are known to bind to TLR ligands. CD14, for example, is able to bind LPS, PGN (85, 86), Pam3CSK4 (87), polyI:C (88), and CpG DNA (89). They also play a role in ligand discrimination, as loss of CD36 impairs the TNFα response against LTA and the TLR2/6 ligand MALP2, but not Pam2CSK4, Pam4CSK4, LPS, PGN, zymosan, polyI:C, or CpG DNA (90). MD2 has also been shown to associate with both TLR4 and TLR2 to enhance signaling by their cognate ligands (91). It is unknown whether binding of these accessory proteins changes the conformation of the intracellular domains of TLRs and, if so, how this affects signaling. The use of peptides mimicking the intracellular domains of TLRs offers a promising platform to study TLR dimerization and receptor assembly (28).

Although PI3K has been shown to regulate the balance of pro- vs. anti-inflammatory responses following TLR4 activation, the precise regulatory role of PI3K in the regulation of pro- vs. anti-inflammatory signaling from other TLRs, and specifically from TLR2, remains untested. While PI3K-dependent regulation of TLR4 anti-inflammatory signaling has been shown to involve differential compartmentalization of the TLR complex, TLR2 only induces TNFα and type I IFN from endosomal compartments (56, 92). Like TLR4, TLR2 also uses the MAL adaptor molecule, and activation of TLR2/6 with diacylated lipopeptides has been shown to lead to MAL-p85α interaction and Akt-mediated macrophage polarization, suggesting a potential mechanism for TLR2 signaling plasticity (93). However, for TLR2, the regulatory role of PI3K may not involve differential compartmentalization of signaling complexes (Peres and Madrenas, unpublished observations).

**Figure 2:**
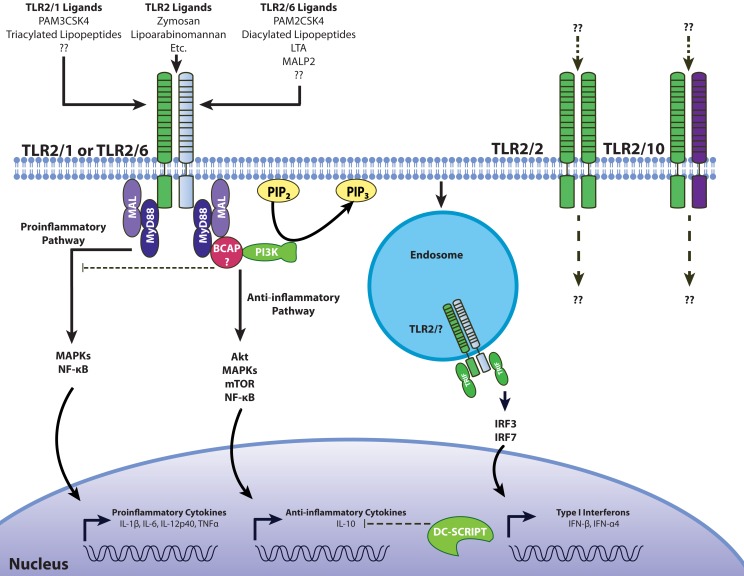
**Model for pro- and anti-inflammatory signaling through TLR2**. Like TLR4, pro-inflammatory signaling through TLR2 involves the recruitment of the adaptors MyD88 and MAL/TIRAP to induce inflammatory cytokines through ERK, JNK, and NF-κB. BCAP interacts with both MyD88 and MAL to recruit PI3K, which then phosphorylates Akt, inducing the secretion of anti-inflammatory IL-10. The involvement of DC-SCRIPT in suppressing TLR2-depdendent IL-10 production has not been tested. TLR2 activation can also induce type I interferons through IRF3 and IRF7 from endosomal compartments (38, 92). Differences in signaling between the TLR2/1 and TLR2/6 heterodimers, as well the potential involvement of hypothesized TLR2/2 and TLR2/10 receptor conformations presents another challenge in understanding the plasticity of TLR2-dependent responses.

Sustained ligation of TLR2 with TLR1, TLR6, or TLR10 brings the TIR domains within close proximity, forming the platform for TIR adaptors, and leads to downstream signaling events. Availability of intracellular adaptors is another factor that can accommodate different signaling patterns from TLR2. For anti-inflammatory responses, we already mentioned that TLR2/6 heterodimers can use the MAL adaptor molecule leading to PI3K-Akt activation, which includes IL-10 production among its functional correlates (93). As previously mentioned, certain macrophage populations have been shown to induce IFN-β through a TLR2 and TRIF-dependent mechanism (38). Type I IFN have been suggested as initiators of the IL-10 response, but it is not clear if there is a mechanistic link. However, PBMCs stimulated with heat killed *S. aureus* may not require type I IFN for the IL-10 response (Peres and Madrenas, unpublished observations). Another adapter linked to TLR-induced PI3K activation is the B-cell adapter for PI3K (BCAP). It has been shown that the TIR domain of BCAP interacts with MyD88 and MAL to suppress production of pro-inflammatory cytokines in bone-marrow-derived macrophages (94, 95) (Figure 2). Mice deficient in BCAP showed impaired Akt phosphorylation in response to stimulation with LPS, CpG, and Pam3CSK4 (94), but the phosphorylation of the MAP kinases extracellular-signal regulated kinase (ERK) and JNK in response to LPS was not affected (95).

Interestingly, Ni et al. (95) found that IL-10 production after LPS stimulation did not differ between the BCAP-deficient and control groups, seemingly contradicting the findings in p110δ enzymatically deficient mice (2). Further investigation is required on the possible differential role of PI3K in TLR4- and TLR2-mediated anti-inflammatory responses. Since PI3K constitutes a relatively large family of enzymes comprising three classes, each with several isoforms that serve diverse cellular functions, one cannot rule out that the plasticity in TLR signaling results, in part, from different usage of these isoforms. Use of chemical inhibition of the PI3K/Akt pathway has its limitations. For example, the often-used wortmannin is a broad PI3K inhibitor that may impact other cellular functions. Therefore, further investigation is required to determine the mechanistic role of different PI3K isoforms in the TLR2-dependent anti-inflammatory response.

Toll-like receptor 2 signaling plasticity can also result from the specific stage of differentiation of the cell expressing this receptor. The main cell candidate responsible for the IL-10 response in human PBMCs is the CD14^+^ monocyte. Experiments using *in vitro* differentiation of primary monocytes into macrophages and DCs indicate that macrophages, not DCs are the primary mediators of the immunomodulatory or anti-inflammatory response (71). Recently, a potential mechanism has been hypothesized that could explain this difference in TLR2-dependent IL-10 secretion. DC-SCRIPT is a transcription factor found in all DC subsets, including *in vitro* monocyte-derived DCs, myeloid DCs, plasmacytoid DCs, and Langerhans cells, but is not expressed in other leukocytes (96). When DC-SCRIPT was knocked down using siRNA, the IL-10 levels were increased compared to controls in response to stimulation with LPS and R848, a TLR7/8 ligand (97). In contrast, the IL-10 response to poly I:C was unchanged (98). Unfortunately, TLR2 ligands were not examined in this study. DC-SCRIPT is a negative regulator of IL-10 production following activation of certain TLRs and represents a plausible explanation for the differences observed between monocyte-derived macrophages and DCs. Further investigation is required to characterize anti-inflammatory TLR2 responses in both monocyte-derived DCs and *in vivo* DC populations.

Considering all the factors mentioned above, we propose that the plasticity of TLR2 signaling is a reflection of the evolving nature of the bacterial cell wall. As a result of the selective pressure exerted on the microbes by the interacting hosts, the array of ligands for TLR2 increases. The versatility accumulated by the immune system over the phylogeny of the species is thus revealed by the interaction with these ligands. The outcome of these interactions may involve and, indeed, favor the microbial capacity to induce anti-inflammatory responses not only as an immune evasion mechanism, which ultimately may eliminate the host and negatively impact the microbe, but also as a way to promote pathobiosis or even commensalism.

## Therapeutic Potential of TLR2 Signaling Plasticity

The evolving nature of MAMPs upon host-imposed selective pressure offers opportunities to explore novel immune modulatory mechanisms with therapeutic potential. The importance of TLR-induced anti-inflammatory pathways has been corroborated *in vivo*. For example, chronic recurrent multifocal osteomyelitis (CRMO) is an autoimmune bone disorder, and patients were shown to have impaired IL-10 expression downstream of TLR4 (99). Hofmann et al. (99) showed that CRMO patients have deficient ERK 1 and 2 signaling following TLR4 activation. This results in an increase in the TNFα/IL-10 ratio, providing a possible mechanism for the pathogenesis of CRMO and suggests novel therapeutic targets. TLR2-dependent anti-inflammatory signaling may prove to be equally important in other clinical settings.

The lipopeptides that can act on TLR2 have garnered much attention as tools to understand the immune system and also as potential adjuvants because of the feasibility of their synthesis in the laboratory setting. Much of our understanding of TLR2 signaling comes from the use of the synthetic lipopeptides, Pam3CSK4, and Pam2CSK4, but it is unknown to what extent these ligands recapitulate the binding of and responses to physiological ligands. Pam3CSK4 and Pam2CSK4 were used to structurally characterize the binding of TLR2/1 and TLR2/6 to their cognate ligands (66, 67). Using x-ray crystallography, the binding profiles of human TLR2 were identified. TLR2 recognizes two acyl moieties on Pam2CSK4 or Pam3CSK4 while TLR1 recognizes one acyl moiety, and TLR6 does not have a binding pocket for any acyl chains. It is interesting to note that the accessory molecule CD14 has been reported to bind Pam3CSK4 and other triacylated lipopeptides and induce the formation of TLR2/1 signaling complexes (87). Presently, this finding has not been reported with Pam2CSK4. In addition, LPS binding protein (LBP) appears to play an independent and redundant role in Pam3CSK4 or bacterial lipoprotein presentation to the TLR2 signaling complex (100). Intriguingly, Pam2CSK4 signaled independently of TLR6 in murine bone marrow-derived macrophages and B lymphocytes (69). Currently, there is still no single defined natural ligand for TLR2. Furthermore, the differences in PRR signaling between mice and humans as well as ambiguities in potential ligands have yet to be elucidated. For now, it is believed that bacterial lipoproteins are the family of biomolecules responsible for TLR2 signaling (64).

Synthetic lipopeptides have also been examined as adjuvants (101). Pam3CSK4 showed promise for live-attenuated vaccines as it enhanced infection of paramyxoviruses *in vitro* and *in vivo* (102) as well as RSV infection in multiple cell types. However, the related lipopeptides Pam-Cys-SK4 and PHCSK4 did not activate TLR signaling although they were able to enhance the binding and infection of APCs by the virus, even though RSV primarily targets airway epithelial cells (103). The enhanced infectivity may allow live-attenuated vaccines to produce a more robust immune response and confer significantly stronger secondary responses (102, 103). In this regard, Pam-Cys-SK4 and PHCSK4 could be useful in the development of vaccines.

Synthetic lipopeptides targeting TLR2 have also been considered as potential modulators of T cell subset differentiation, which may be a strategy to confer protection to certain chronic inflammatory, immune mediated diseases or to enhance vaccine efficacy (104). For example, Pam3CSK4 enhanced CD8^+^ regulatory T cell (T_reg_) survival (105). Interestingly, glycolipopeptide constructs can be used to design composite tumor vaccines (106). These compounds contain carbohydrate antigens, CD4^+^ or CD8^+^ T cell epitopes, and TLR2 stimulating lipid chains. By compounding these properties, improved vaccines against malignant cancer cells may be generated. Also, by introducing structural modifications in native ligands it should be possible to enhance the anti-inflammatory properties of TLR2 signaling (107).

Finally, a role for TLRs (specifically TLR2) and other PRRs is emerging in the crosstalk between the host and its microbiome. TLR2 has been linked to the establishment and regulation of the microbiota in different sites. One such site is the skin, where staphylococcal LTA has been shown to down-regulate inflammatory cytokine release by keratinocytes in a TLR2-dependent manner (5). Another site is the gut, where microbiota has been shown to play an active role in the maturation and homeostasis of the host immune system (108, 109). Studies conducted on *Bacteroides fragilis*, a ubiquitous gut microbe, revealed that polysaccharide A (PSA) was involved in correcting CD4^+^ T cell deficiency and the T_H_1/T_H_2 imbalance in germ-free mice (109). The significance of this mechanism has been demonstrated in disease models, where experimental colitis induced by *Helicobacter hepaticus* was more severe in *B. fragilis* ΔPSA compared to wild-type *B. fragilis*. Moreover, purified PSA protects animals from experimental colitis through an IL-10 dependent mechanism (110). PSA was found to signal through TLR2 directly on T_reg_ cells to promote immune tolerance by suppressing T_H_17 responses (6). It has been suggested that PSA is a member of a new class of TLR ligands known as symbiont-associated molecular patterns (SAMPs) where TLR signaling paradoxically may allow persistent colonization. These effects may not be limited to *B. fragilis* but be shared by other components of the microbiome (e.g., clostridia) (111, 112).

The immunological environment in the gut and other sites where the host interacts with its microbiome is a critical site for host-microbe co-evolution and dynamic selection of MAMPs and PRRs. There is an advantage for the host to evolve sophisticated mechanisms to differentially respond to molecular patterns associated with symbiosis or pathogenicity. The involvement of TLR2 in regulating the gut microbiota and preventing dysbiosis may be just one example of the complexity of TLR ligand co-evolution, providing additional impetus for further investigation of the plasticity of TLR2 signaling and potential avenues to exploit it.

## Conclusion

While the pro-inflammatory responses activated upon detection of Gram-negative and Gram-positive bacteria by TLRs have been well characterized, the TLR-triggered anti-inflammatory responses are only beginning to be elucidated. The current literature regarding these new responses focus on TLR4 but TLR2 is especially interesting to study in this context given the diversity in receptor complexes and the crosstalk between downstream signaling cascades. The challenges ahead are not trivial: (i) the array of ligands for TLR2 may be larger than originally suspected, and its characterization may be an ongoing exercise as suggested by the evidence supporting their evolving nature; (ii) the relevance of various TLR2 ligands in the context of infection is not clear; and (iii) the tools used to study TLR2 activation both structurally and functionally are limited and may not recapitulate the full range of responses following TLR2 activation *in vivo* in response to bacteria. However, the therapeutic promise shown by synthetic TLR2 ligands as well as the emerging significance of TLR2 in regulating the gut microbiota, among other reasons, justify further investigation into the plasticity of TLR2-mediated MAMP recognition and downstream signaling.

## Conflict of Interest Statement

The authors declare that the research was conducted in the absence of any commercial or financial relationships that could be construed as a potential conflict of interest.
